# Toward the Impact of EFL Teachers' Self-Efficacy and Collective Efficacy on Students' Engagement

**DOI:** 10.3389/fpsyg.2021.744586

**Published:** 2021-09-13

**Authors:** Qiaoqiao Lu, Zarina Mustafa

**Affiliations:** ^1^School of Foreign Languages & Cultures, Guangdong University of Finance, Guangzhou, China; ^2^School of Educational Studies, Universiti Sains Malaysia (USM), George Town, Malaysia

**Keywords:** teacher self-efficacy, teacher collective efficacy, student engagement, education, academic success

## Abstract

Given the centrality of students' engagement in their academic success, considerable attention has been paid to this construct and its potential predictors. Notwithstanding, a limited number of studies have focused on the role of teacher self- and collective efficacy as antecedents of student engagement. Further, no review study has been carried out to illustrate the impact of EFL teacher' efficacy on learning engagement. Hence, the current study intends to review the previous studies conducted on this topic to probe into the beneficial effects of EFL teachers' sense of efficacy on students' academic engagement. The predictability power of EFL teachers' self- and collective efficacy was confirmed through empirical and theoretical evidence. The conclusion and pedagogical implications of the finding are also discussed.

## Introduction

Owing to the fact that students' engagement plays a vital role in increasing their learning outcomes (Carver et al., 2021), inspiring students to become involved in the learning process has always been a priority for teachers in all academic contexts. Student engagement is conceptualized as “one's tendency to be behaviorally, emotionally, and cognitively involved in academic activities” (Sharkey et al., 2008, p. 404). As put forward by Appleton et al. (2008), engaged students are those who perceive the learning process positively and put more effort into achieving the course materials. Concerning the importance of student engagement in educational contexts, Wang et al. (2011) submitted that student engagement is tied with higher achievement, continual development, and academic success, mainly due to the fact that engaged students demonstrate more perseverance and effort in pursuing different phases of learning. As such, identifying internal (i.e., student-related factors) and external factors (teacher-related factors, context-related factors) that are capable of predicting students' engagement in instructional-learning contexts is of high importance. In this regard, several studies have been carried to examine the role of student-related factors (e.g., Skinner and Pitzer, 2012; Yin and Wang, 2016; Zhen et al., 2017) and context-related factors (e.g., Chong et al., 2010; Raftery et al., 2012; Chen et al., 2019; Teng and Wang, 2021) in students' level of engagement. Additionally, some empirical and theoretical bodies of research have been dedicated to the role of teacher-related factors in students' learning engagement (e.g., Gibbs and Powell, 2012; Van Uden et al., 2013, 2014; Dewaele and Li, 2021; Jiang and Zhang, 2021; Zheng, 2021). However, a significant portion of studies on teacher-related factors have investigated the effects of teacher interpersonal factors on students' engagement, hence, the role of teachers' personal factors such as self-efficacy and collective efficacy as potential antecedents of student learning engagement has remained elusive in educational research.

One of the important teacher personal factors is teacher self-efficacy, which refers to “teachers' beliefs about their personal capabilities to perform their duties in the classroom” (Klassen et al., 2010, p. 466). As put forward by Stephens (2015), self-efficacious teachers are able: (a) to devise and employ alternative English teaching methods when the desired learning outcomes are not achieved; and (b) to cope with a challenging situation by manipulating the situation's emotional and cognitive processes. In contrast, teachers with low levels of self-efficacy are inclined to dwell on their inadequacies and overestimate the difficulty of challenging situations. When it comes to the significance of EFL teacher' self-efficacy, there is a large consensus among the scholars that self-efficacious teachers are more capable of motivating their pupils to become involved in the learning process (Martin et al., 2012; Van Uden et al., 2013, 2014; Papa, 2015). Another prime instance of teacher personal factors is teacher collective efficacy, referring to “the beliefs teachers possess in their collective capabilities to influence the lives of their students” (Klassen et al., 2010, p. 465). According to Khong et al. (2017), teachers' positive perceptions regarding faculty members' capability to fulfill their professional responsibilities can favorably impact students' engagement, achievement, and academic success.

Despite the significance of teachers' sense of efficacy (i.e., individual and collective efficacy) in enhancing student engagement (Papa, 2015; Khong et al., 2017), only a few studies have been carried out to probe into the association between these variables. Moreover, to our knowledge so far, no review study has been done to elaborate on the definitions of EFL teacher' self-efficacy, teacher collective efficacy, and student engagement, as well as the association between these constructs. In view of the factors afore-mentioned, the current study attempts to fill this gap by reviewing the existing definitions of teacher efficacy and student engagement and highlighting the positive connection between these valuable constructs.

### Student Engagement

Given the complexity and multidimensionality of “*Student Engagement*,” scholars described this concept in various ways. Hu and Kuh (2002), for instance, simply defined student engagement as the amount of effort students dedicate to learning English tasks. Skinner et al. (2009) further conceptualized student engagement as “the quality of students' participation or connection with the educational endeavor and hence with activities, values, individuals, aims, and place that comprise it” (p. 496). In a more comprehensive definition, Zepke and Leach (2010) characterized student engagement as “one's cognitive investment in, active participation in, and emotional commitment to his/her learning” (p. 169).

In an attempt to characterize different dimensions of student engagement, Schaufeli et al. (2002) broke this construct into three main components of “*Vigour*,” “*Dedication*,” and “*Absorption*.” Vigour is referred to the amount of perseverance and effort students demonstrate in executing their academic responsibilities. Dedication, as the second component, is tied with students' sense of pride, enthusiasm, and inspiration for participating in classroom activities. Finally, absorption is related to students' feeling of being thoroughly immersed in learning tasks/activities. In a different classification, Fredricks et al. (2004) categorized the concept of student engagement across three dimensions of “*Cognitive*,” “*Behavioral*,” and “*Emotional*.” According to Fredricks et al. (2004), students' cognitive engagement is intertwined with their tendency and inclination to learn complicated issues. To them, students' behavioral engagement is related to their active and continuous participation in academic activities. Emotional engagement, as the last dimension, relates to students' positive reactions to their classmates, instructors, and learning environment (Fredricks et al., 2004).

### Teacher Self-Efficacy

The concept of self-efficacy refers to “one”s beliefs in his/her capability to organize and execute the courses of action required producing given attainment” (Bandura, 1977, p. 193). More specifically, “*Teacher Self-efficacy*” is characterized as teachers' personal beliefs about their potential to accomplish their academic responsibilities (Klassen et al., 2014). That is, self-efficacious teachers are those who believe in themselves and their professional capabilities. As put forward by Sarfo et al. (2015), the construct of teacher self-efficacy encompasses three major dimensions of “*efficacy for student engagement*,” “*efficacy for instructional strategies*,” and “*efficacy for classroom management*.” As such, self-efficacious teachers are more successful at engaging students, employing instructional strategies, and managing classroom environment (Sarfo et al., 2015).

### Teacher Collective Efficacy

The concept of collective efficacy is generally defined as “a group's shared belief in its conjoint capabilities to organize and execute the courses of action required producing given levels of attainments” (Bandura, 1997, p. 477). Similarly, “*Teacher Collective Efficacy*” refers to teachers' conviction in the collective capacity of faculty members to positively affect students' learning outcomes (Goddard et al., 2015). According to Chong et al. (2010), school and university administrators can enhance teachers' sense of collective efficacy. That is, educational institutions whose instructors demonstrate higher sense of collective efficacy may have more supportive administrators.

### The Effects of Teachers' Self- and Collective Efficacy on Students' Engagement

In an attempt to illustrate the significance of teachers' self- and collective efficacy, Papa (2015) stated that efficacious teachers who believe in their own and their group's professional capabilities are more inclined to implement new instructional methods and approaches which encourage students to take part in classroom activities. In another attempt, Stephens (2015) explicated that teachers with a stronger sense of academic efficacy are more inclined “to engage in pedagogy that is characterized by positive, proactive, and solution-focused orientations, resulting in increased student engagement” (p. 2). Similarly, Van Uden et al. (2013) postulated that instructors' sense of efficacy can favorably influence their “*affective orientation*” toward their pupils, leading to higher student engagement. Furthermore, Sarfo et al. (2015) also proposed that efficacious teachers commonly exhibit higher persistence and effort, which inspire students to become engaged in the learning process.

## Empirical Studies

Given the pivotal function of teachers in enhancing students' engagement (Stephens, 2015), several scholars have attempted to examine the effects of teacher-related factors on learning engagement. However, the majority of these studies have focused on teachers' interpersonal factors and their associations with student engagement (e.g., Derakhshan, 2021; Zhang, 2021; Zheng, 2021). Hence, a small number of studies have explored the impact of teacher personal factors such as self- and collective efficacy on students' academic engagement (e.g., Van Uden et al., 2013, 2014; McDavid et al., 2018). Van Uden et al. (2014) studied the role of teachers' beliefs about personal and collective capabilities in enhancing students' engagement. In doing so, 200 teachers and 2,288 took part in this study. Employing digital questionnaires, the participants' viewpoints and attitudes toward the association between teachers' sense of efficacy and learning engagement were gathered. The analysis of participants' responses revealed that teacher efficacy can dramatically and positively predict students' learning engagement. In a similar vein, McDavid et al. (2018) investigated teachers' perceptions regarding the function of their self-efficacy in their students' academic engagement. To do so, 148 faculty members were asked to complete some online questionnaires. Analyzing participants' responses to the questionnaires, the researchers found a favorable association between learning engagement and teachers' sense of efficacy. That is, participants perceived teacher sense of efficacy as a strong antecedent of students' academic engagement.

## Conclusion and Pedagogical Implications

In this review study, the constructs of teacher self-efficacy, collective efficacy, and student engagement were thoroughly characterized. Further, the effects of teachers' individual and collective efficacy on students' engagement were illuminated through the use of empirical and theoretical evidence. With regard to the existing evidence, it can be inferred that teachers' sense of individual and collective efficacy can positively influence students' learning engagement. However, it is worth noting that teachers' individual as well as collective efficacy have been neglected in enhancing students' learning engagement. To some extent, this finding can be illuminative and inspiring for both pre- and in-service teachers in any educational institution (i.e., school, university, etc.). Given the significance of teachers' individual and collective efficacy in fostering students' engagement (Stephens, 2015), teachers who aspire to increase their students' engagement should believe in their own and their colleagues' professional capabilities. Additionally, this review study has an important implication for administrators. As put forward by Chong et al. (2010), supportive administrators are able to dramatically enhance teachers' sense of collective efficacy. As such, educational administrators are expected to support their teachers in improving their collective efficacy, which is essential for increased learning engagement. Moreover, researchers can continue conducting studies on the role of self-efficacy, collective efficacy, and other teacher-student interpersonal variables (Fathi et al., 2020; Xie and Derakhshan, 2021).

## Author Contributions

QL and ZM read the relevant literature and illuminated the effects of teachers' individual, and collective efficacy on students' engagement.

## Funding

National Educational Science 13th Five-Year Plan National Key Project≪Research on the Issues and Institutional Innovation of Educational Integration Development in Guangdong-Hong Kong-Macao Greater Bay Area≫ (AGA200015). 2021 Guangdong Province Science Philosophy and Social Science Planning Project≪Research on Practice Patterns and Circular Strategies of Higher Education Internationalization in the Guangdong-Hong Kong-Macao Greater Bay Area from the perspective of higher education cluster development≫. 2021 Guangdong Province Educational Science Planning Project≪Research on the Path to Construct Applied Foreign Language Talents in the Guangdong-Hong Kong-Macao Greater Bay Area from the perspective of the development of higher education clusters≫.

## Conflict of Interest

The authors declare that the research was conducted in the absence of any commercial or financial relationships that could be construed as a potential conflict of interest.

## Publisher's Note

All claims expressed in this article are solely those of the authors and do not necessarily represent those of their affiliated organizations, or those of the publisher, the editors and the reviewers. Any product that may be evaluated in this article, or claim that may be made by its manufacturer, is not guaranteed or endorsed by the publisher.
